# Enhanced generation of human induced pluripotent stem cells by ectopic expression of Connexin 45

**DOI:** 10.1038/s41598-017-00523-y

**Published:** 2017-03-28

**Authors:** Qiong Ke, Li Li, Xin Yao, Xingqiang Lai, Bing Cai, Hong Chen, Rui Chen, Zhichen Zhai, Lihua Huang, Kai Li, Anbin Hu, Frank Fuxiang Mao, Andy Peng Xiang, Liang Tao, Weiqiang Li

**Affiliations:** 1 Program of Stem Cells and Regenerative Medicine, Affiliated Guangzhou Women and Children’s Hospital, Zhongshan School of Medicine, Sun Yat-Sen University, Guangzhou, 510623 China; 2 Center for Stem Cell Biology and Tissue Engineering, Key Laboratory for Stem Cells and Tissue Engineering, Ministry of Education, Sun Yat-Sen University, Guangzhou, 510080 China; 3 Department of Biology, Zhongshan School of Medicine, Sun Yat-Sen University, Guangzhou, 510080 China; 4 Department of Pharmacology, Zhongshan School of Medicine, Sun Yat-Sen University, Guangzhou, 510080 China; 5 Lung Biology Laboratory, Department of Medicine, Division of Pulmonary, Allergy and Critical Care, Columbia University Medical Center, New York, New York 10032 USA; 6 Guangdong Key Laboratory of Reproductive Medicine, Guangzhou, Guangdong 510080 China; 7 Center for Reproductive Medicine, Key Laboratory for Reproductive Medicine of Guangdong Province, The Third Affiliated Hospital of Guangzhou Medical University, Guangzhou, 510140 China; 8 Department of Ultrasound, the Third Affiliated Hospital of Sun Yat-sen University, Guangzhou, 510632 China; 9 Department of General Surgery, the First Affiliated Hospital of Sun Yat-sen University, Guangzhou, 510080 China; 10 State Key Laboratory of Ophthalmology, Zhong Shan Ophthalmic Center, Sun Yat-sen University, Guangzhou, 510060 China; 11 Department of Biochemistry, Zhongshan School of Medicine, Sun Yat-Sen University, Guangzhou, 510080 China

## Abstract

Somatic cells can be successfully reprogrammed into pluripotent stem cells by the ectopic expression of defined transcriptional factors. However, improved efficiency and better understanding the molecular mechanism underlying reprogramming are still required. In the present study, a scrape loading/dye transfer assay showed that human induced pluripotent stem cells (hiPSCs) contained functional gap junctions partially contributed by Connexin 45 (CX45). We then found CX45 was expressed in human embryonic stem cells (hESCs) and human dermal fibroblasts (hDFs) derived hiPSCs. Then we showed that CX45 was dramatically upregulated during the reprogramming process. Most importantly, the ectopic expression of CX45 significantly enhanced the reprogramming efficiency together with the Yamanaka factors (OCT4, SOX2, KLF4, cMYC - OSKM), whereas knockdown of endogenous CX45 expression significantly blocked cellular reprogramming and reduced the efficiency. Our further study demonstrated that CX45 overexpression or knockdown modulated the cell proliferation rate which was associated with the reprogramming efficiency. In conclusion, our data highlighted the critical role of CX45 in reprogramming and may increase the cell division rate and result in an accelerated kinetics of iPSCs production.

## Introduction

Somatic cells, such as human fibroblasts, can be successfully reprogrammed into pluripotent stem cells by ectopically expressing defined pluripotency-related transcriptional factors^1–3^. This induced pluripotent stem cell (iPSC) technology provides a useful platform for pathogenesis studies and drug screening by using human patient-specific pluripotent stem cell lines for modelling disease processes in vitro^4–7^. It also raises the possibility of clinical application of personalized stem cell-based therapies while avoiding the immune rejection as well as ethical concerns. Although great progress has been made in this field, iPSC technology is still limited by its low efficiency. Further exploration of the molecular mechanisms underlying reprogramming may facilitate the development of high-quality and efficient methods of iPSC generation.

Gap junction (GJ), an important intercellular communicating junction, is made up of two connexons, which are composed of six transmembrane proteins, termed connexins (CX)^8, 9^. Gap junctional intercellular communication (GJIC) refers to the diffusion and exchange of intracellular molecules of less than 1–1.5 kDa (i.e., small ions, second messengers, amino acids, metabolites, and peptides, etc.) between neighboring cells and involves in the regulation of diverse cellular processes^10–15^.

To date, at least 21 members of the CX gene family have been reported in the human genome^16^. Wong et al. demonstrated that functional GJIC was characteristically present in undifferentiated human embryonic stem cells (hESCs)^17^. Transcripts encoding 18 CX isoforms are expressed by hESCs and only a few CXs, such as CX43, CX45, and CX40, have been confirmed at protein level^18, 19^. Previous studies have reported that CX43 and CX45 mRNAs are highly enriched in hESCs compared to a range of somatic tissues or spontaneously differentiated hESCs as detected by microarray analysis^20, 21^. Several studies confirm the knockdown of CX expression in mouse ESCs reduces cell proliferation and downregulates the expression of pluripotency markers^22^. Such data demonstrated that CX contributes substantially an essential role in maintaining ESCs in the undifferentiated state.

During the reprogramming, single cells gather together and finally form compact colonies with tight cellular association as ESCs-like state. Huang et al. reported that adherens junctions, GJs, focal adhesions and tight junctions were involved in complicated intercellular crosstalk that occurs during reprogramming^23^. So we hypothesize that GJ may play a crucial role in the generation of iPSCs. Sharovskaya et al. had reported that GJIC in incompletely reprogrammed cells was decreased compared with that in the somatic cells, but GJIC in completely reprogrammed cells exceeded that in the somatic cells and was comparable to that in hESCs^24^. But they did not mention the functions of CXs in the reprogramming process. Although important roles of CX expression and/or GJIC in ESCs/iPSCs can be currently perceived, many critical questions including precise mechanisms by which CX expression influences pluripotency and reprogramming remain to be clarified. Our previous report confirmed that CX43 is involved in the generation of hiPSCs but the roles of the other CXs reprogramming process are still unknown.

Here, we demonstrate that CX45 is highly enriched in hDFs-derived undifferentiated hiPSCs but absent in hDFs and CX45 contributes to functional GJIC in hiPSCs. We also find that CX45 expression is dramatically upregulated during the reprogramming process. Enhanced iPS cell generation can be achieved by overexpression of CX45, while the knockdown of CX45 results in reduced reprogramming efficiency. Further mechanistic study indicates that either CX45 overexpression or knockdown affects the cell proliferation by changing p21 and cyclin D1 expression.

## Results

### CX45 contributes to GJIC function in human iPSCs

Adult human dermal fibroblasts (hDFs) derived hiPSCs were generated and characterized as previously described^1^. Both the *in vivo* and *in vitro* analyses revealed that these hiPSCs exhibited the similar characteristics of hESCs, particularly the capacities of self-renewal and differentiation.

**Figure 1:**
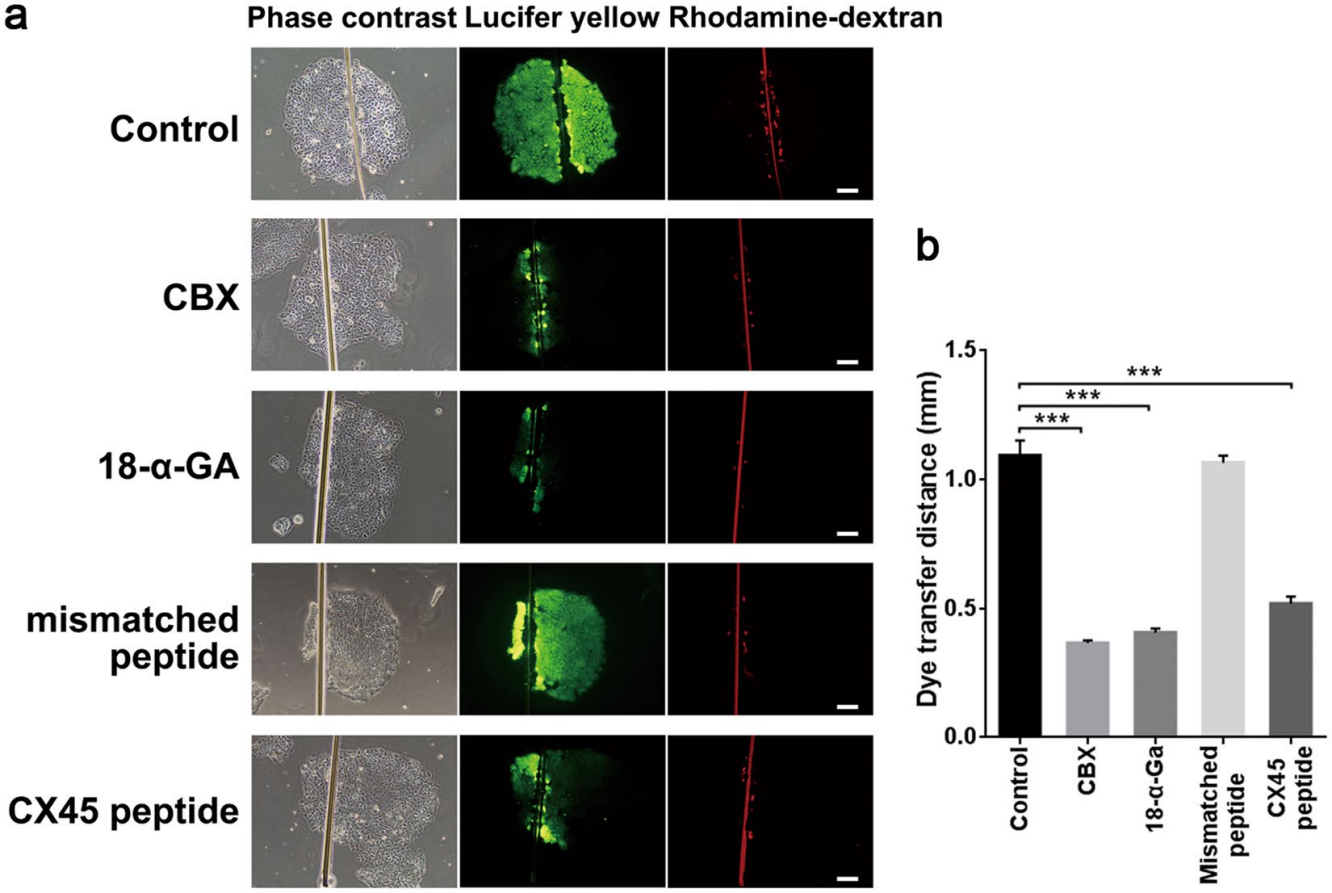
GJIC in human iPSCs. (**a**) Phase-contrast and fluorescence micrographs with Lucifer yellow and rhodamine-dextran. Human iPS cells were incubated in the absence or presence of CBX, 18-α-GA, the mismatched mimetic peptide and CX45 mimetic peptide. Scale bar = 200 μm. (**b**) The histogram shows an analysis of the distance between the dye transfer front and the scrape line of each group. The data are presented as the mean ± S.E.M. (n = 3) and are representative of three independent experiments. ***P < 0.001 versus the control.

We next evaluated functional coupling among hiPSCs using the scrape loading/dye transfer assay. As shown in Fig. 1a, confluent cultures were scraped and incubated with the fluorescent dye Lucifer yellow (LY; green; gap junction-permeable) and the fluorescent dye rhodamine-dextran (RD; red; gap junction-impermeable). Extensive diffusion of LY was observed throughout the hiPSC colonies, and the average transfer distance of LY was approximately 1.09 ± 0.12 mm (mean ± S.E.M.). Two special gap junction blocker carbenoxolone (CBX) and 18-α-glycyrrhetinic acid (18-α-GA) were used to confirm the existence of gap junction communication among iPSCs. Both of the CBX and 18-α-GA significantly reduced the transfer of LY among cells; the LY transfer distances were approximately 0.37 ± 0.02 mm (mean ± S.E.M.) and 0.41 ± 0.03 mm (mean ± S.E.M.), respectively. To further show whether CX45 contributes to this function or not, a peptide specifically blocked CX45 formed GJ was used, as shown in Fig. 1b it could partially reduce the dye transfer (mean ± S.E.M.: 0.52 ± 0.06 mm) compared with the transfer level observed in control. The mismatched mimetic peptide as a negative control had no effect and the dye transfer in this group (mean ± S.E.M.: 1.06 ± 0.06 mm) was similar to control group. By contrast, the gap junction-impermeable fluorescent dye RDs were retained within the scratch in all the groups. Taken together, these results suggest that hiPSCs are coupled through functional gap junctions and that CX45 contributes to this function.

### Increased CX45 expression during reprogramming

**Figure 2:**
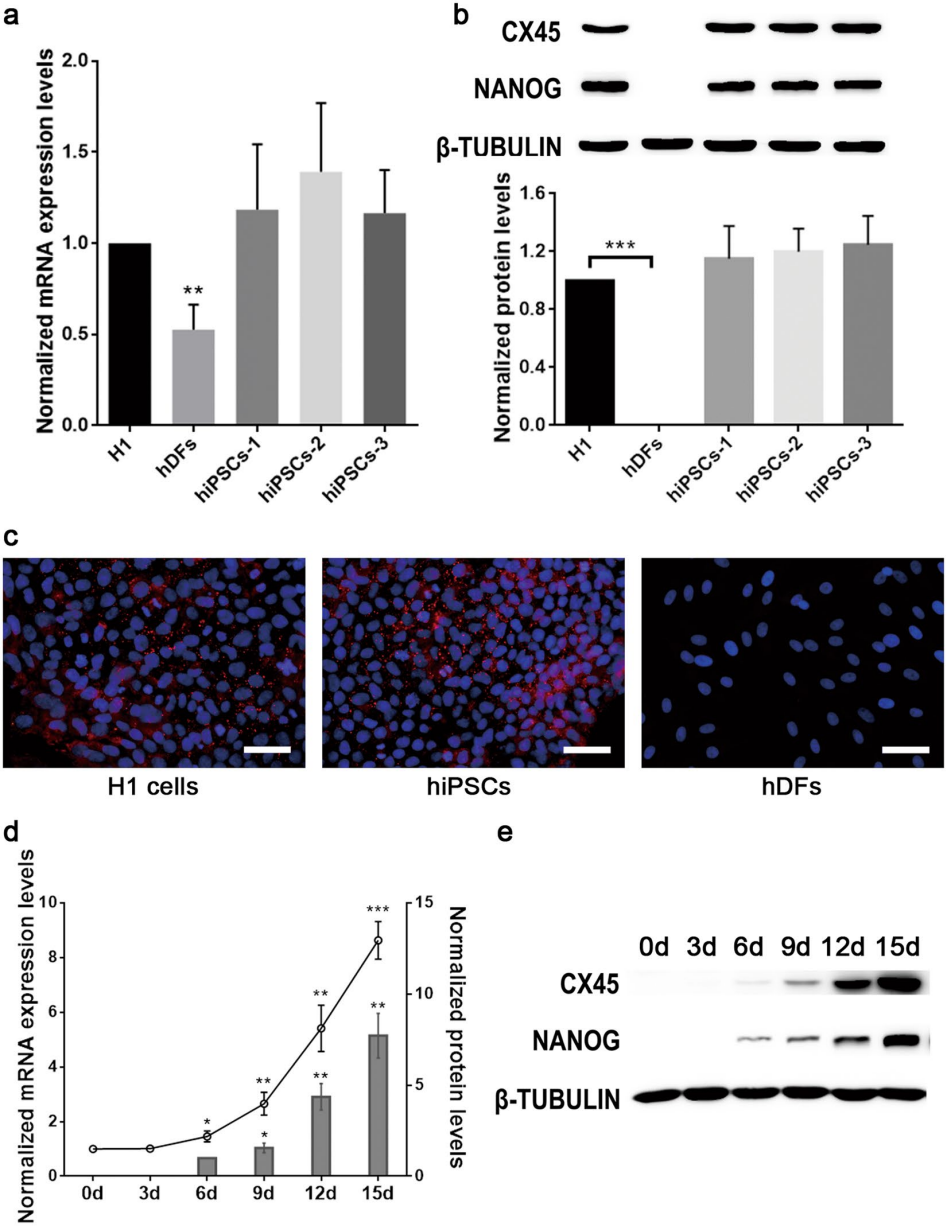
CX45 expression increased in OSKM transduced hDFs during the reprogramming process. (**a**) CX45 expression in H1 cells, hDFs and hiPSCs was assessed by real-time PCR; CX45 expression in the H1 cells was arbitrarily assigned a value of 1.0. (**b**) CX45 protein level in H1 cells, hDFs and hiPSCs was assessed by western blotting analysis; CX45 protein level in the H1 cells was arbitrarily assigned a value of 1.0. The data are presented as the mean ± S.E.M. (n = 3) and are representative of three independent experiments. **P < 0.01, ***P < 0.001 versus H1 cells. (**c**) Immunofluorescence analysis of CX45 in H1 cells, hiPSCs and hDFs; the nuclei were visualised with DAPI staining (blue). Scale bar = 50 μm. (**d**) CX45 expression on the indicated days in OSKM transduced hDFs during the reprogramming process was assessed by real-time PCR (dot plot) and western blotting (column graph); CX45 mRNA expression on day 0 was arbitrarily assigned a value of 1.0. CX45 protein levels on day 6 was arbitrarily assigned a value of 1.0. The data are presented as the mean ± S.E.M. (n = 3) and are representative of three independent experiments. *P < 0.05, **P < 0.01, ***P < 0.001 versus the one assigned a value of 1.0. (**e**) CX45 and NANOG expression on the indicated days in OSKM transduced hDFs during the reprogramming process was assessed by western blotting analysis.

In order to detect the gene expression of CX45, we used real-time PCR to detect CX45 mRNA expression levels in these cells. All of the hiPS cell lines (hiPSCs 1, 2, 3) synthesised CX45 mRNAs and their levels were similar to that of hESCs, while CX45 mRNAs level in hDFs was the lowest (Fig. 2a). In cultured cells, the similarly high levels of CX45 protein were identified in these hiPSCs lines and one commercial hESCs (H1 cell line), but it was not detectable in hDFs as assayed by western blotting (the half-life for CX45 is about 3–4 h^25^) (Fig. 2b). CX45 was primarily localized along the borders between neighbouring cells in both the hESCs and hiPSCs as demonstrated by immunofluorescence staining (Fig. 2c).

To further explore the relationship between CX45 expression and the acquisition of pluripotency during the reprogramming process, we collected samples every 3 days until 15 days after the viral transduction of reprogramming factors (OCT4, SOX2, KLF4, cMYC - OSKM) and evaluated the expression levels of both CX45 and the pluripotency marker NANOG. RT-PCR revealed that CX45 expression increased during the period of hiPSCs formation and finally was up to 9 times at 15 days post reprogramming (Fig. 2d). CX45 protein could be first detectable around 6 days after the viral transduction by western blotting along with the pluripotency marker NANOG, and then increased significantly around 12 days post reprogramming (Fig. 2d and e). At day 15 after transduction, the CX45 protein level was nearly 8-fold greater than that on day 6, showing that the CX45 may play an important role in the reprogramming process.

### Ectopic expression of CX45 enhances hiPSC generation

**Figure 3:**
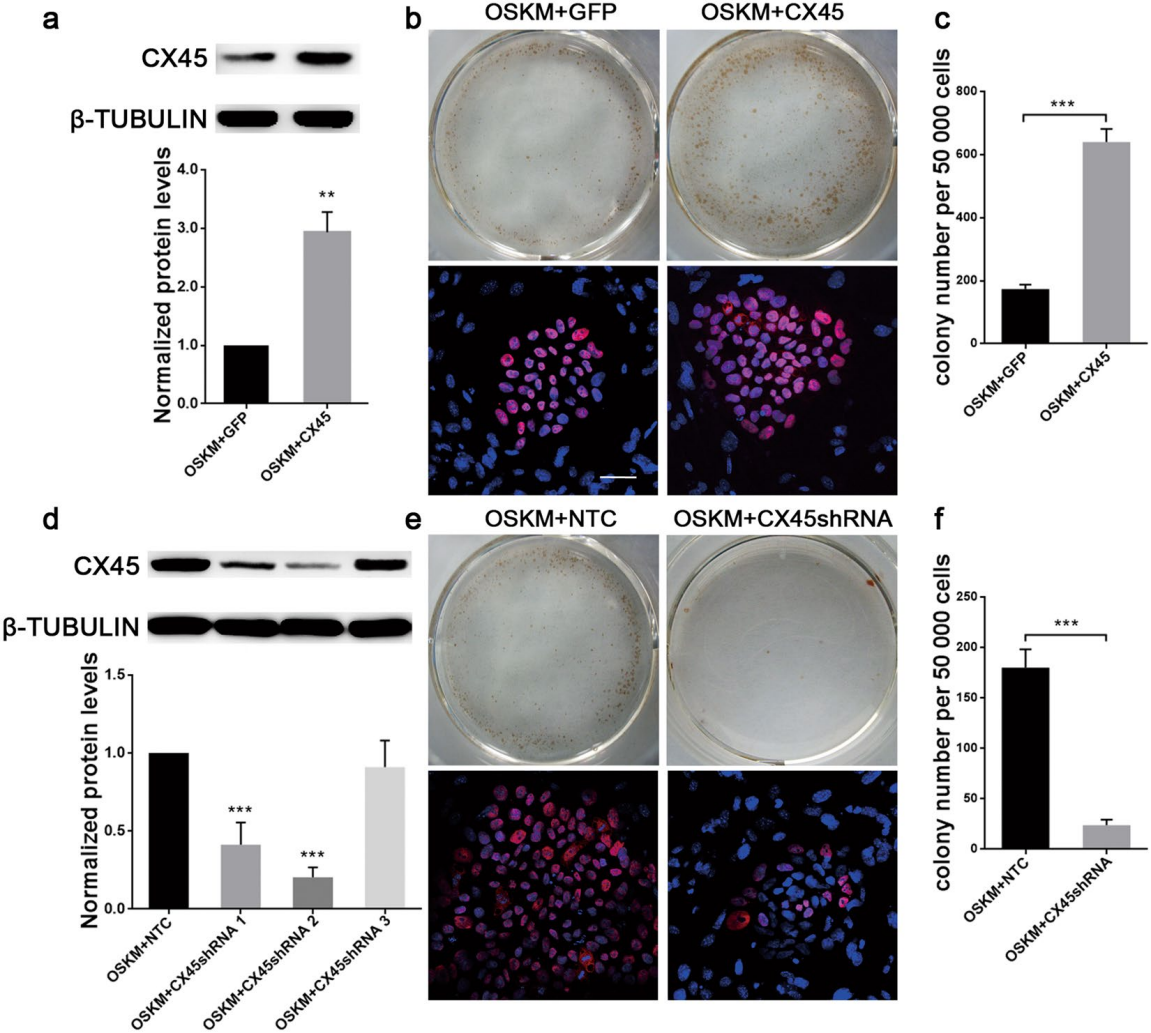
Ectopic expression CX45 promotes the reprogramming efficiency. (**a**) CX45 overexpression on hDFs was analysed by western blotting at day 3. (**b**) Representative images of TRA-1-60-positive (TRA-1-60^+^) colonies (upper) and immunofluorescence analysis of OCT4 (lower) in each group. Reprogramming in OSKM + CX45 group results in more typical TRA-1-60^+^ colonies compared to controls. (**c**) Quantification of TRA-1-60^+^ colonies per 50 000 cells as indicated. (**d**) CX45 expression in hDFs that was lentivirally transduced with CX45 shRNA and OSKM was analysed by western blotting analysis at day 5. (**e**) Representative images of TRA-1-60^+^ colonies (upper) and immunofluorescence analysis of OCT4 (lower) in each group. Ablation of endogenous CX45 by CX45shRNA results in less TRA-1-60^+^ colonies compared to controls. (**f**) Quantification of TRA-1-60^+^ colonies per 50 000 cells as indicated. The data are presented as the mean ± S.E.M. (n = 3) and are representative of three independent experiments. **P < 0.01, ***P < 0.001 versus OSKM + GFP or OSKM+NTC group. OSKM+GFP, eGFP expression vector plus OSKM; OSKM+CX45, CX45 expression vector plus OSKM; OSKM+NTC, containing nonspecific shRNA plus OSKM; OSKM+CX45shRNA1, 2 and 3, containing CX45 shRNA target sequences plus OSKM. Scale bar = 50 μm.

To further investigate the role of CX45 in hiPSC generation, we introduced an ectopic CX45 expression vector in the reprogramming process and tested whether CX45 could promote iPSC generation. pFinal/PGK-puro-EF1α-CX45-IRES-eGFP was constructed as an overexpression vector (abbreviated as CX45, Supplementary Fig. S1a), and the GFP vector pFinal/PGK-puro-EF1α-eGFP (abbreviated as GFP) was used as a negative control. When OSKM plus the GFP vector (OSKM+GFP group) or CX45 expression vector (OSKM+CX45 group) were added to the HDFs, more than 90% of the cells were infected as reflected by eGFP fluorescence at day 2 (Supplementary Fig. S1b). Figure 3a shows that CX45 virus transduction (OSKM+CX45) significantly increases CX45 protein level in hDFs; its expression was approximately 3-fold greater than the control. On day 5, these cells were singularized and plated in ES medium to 6-well plates carrying irradiated feeder layers. On day 28, TRA-1-60 expression was assayed by immunocytochemistry staining and TRA-1-60^+^ colonies were counted by three independent experiments for each group. We obtained 173 ± 13 (mean ± S.E.M.) TRA-1-60^+^ colonies from 50 000 cells in OSKM+GFP group, while the TRA-1-60^+^ colonies in the OSKM+CX45 group were 640 ± 41 (mean ± S.E.M.) from 50 000 cells. Moreover, the colony size was larger and better clonal morphology appeared in OSKM+CX45 group than control. Immunofluorescence staining of pluripotency markers OCT4 showed stronger positive response in the OSKM+CX45 group than the control group. When we repeated the experiment, we still obtained an approximately 4-fold increase in the reprogramming efficiency when counting the TRA-1-60^+^ colonies (Fig. 3b and c). Using another HDFs cell line, similar results were observed as shown in Supplementary Fig. S2a. We got 174 ± 20 (mean ± S.E.M.) TRA-1-60^+^ colonies in OSKM+GFP group and 549 ± 98 (mean ± S.E.M.) TRA-1-60^+^ colonies in OSKM+CX45 group (Supplementary Fig. S2b). But when we reduced the transcription factors, only OSM+CX45 group showed more effective compared with the control group. Further, the picked colonies from OSKM+CX45 group could be maintained *in vitro* and those colonies showed pluripotency and differentiation potential after 6 passages (Supplementary Fig. S3).

### Repression of CX45 expression reduces reprogramming efficiency

We then examined whether ablation of endogenous CX45 could reduce reprogramming efficiency. Three lentiviral vectors encoding CX45 short hairpin RNAs (CX45shRNA1, 2 and 3), were constructed into the pLL3.7 vector for knockdown the expression of CX45. A negative control vector containing nonspecific targeting control shRNA (named NTC) was also constructed. The hDFs transduced with either control or CX45 shRNA lentiviruses plus OSKM, and then the transfection efficiency was reflected by eGFP fluorescence. More than 90% of the hDFs expressing green fluorescent protein could be found in those four groups (Supplementary Fig. S1c). Western blotting showed that CX45shRNA1 and CX45shRNA2 can highly effectively knockdown CX45 protein level (59% and 80%, respectively), while CX45shRNA3 was significantly less effective. No CX45 knockdown was observed in the hDFs treated with NTC vector (Fig. 3d). We then introduced four reprogramming factors into hDFs with NTC or shRNA (OSKM+NTC group and OSKM+CX45shRNA group), respectively. To explore the effect of CX45 knockdown on iPSC generation, we counted the TRA-1-60^+^ colonies after the viral transduction of reprogramming factors (Fig. 3e and f). When CX45 expression was disrupted by shRNA2 after OSKM-induced reprogramming, we obtained only 23 ± 5 (mean ± S.E.M.) TRA-1-60^+^ colonies from 50 000 cells and there were still 180 ± 18 (mean ± S.E.M.) TRA-1-60^+^ colonies in OSKM+NTC group on day 28. Immunofluorescence staining showed that OCT4 positive colonies were hardly found in the OSKM+CX45shRNA group. Similar results were obtained in parallel experiments (approximately 83% decrease of TRA-1-60^+^ colonies after knockdown of CX45) and statistical results showed that there was prominent significance. These results suggest that endogenous CX45 expression may be critical for hiPSC generation.

### CX45 overexpression or knockdown affects the cell proliferation

**Figure 4:**
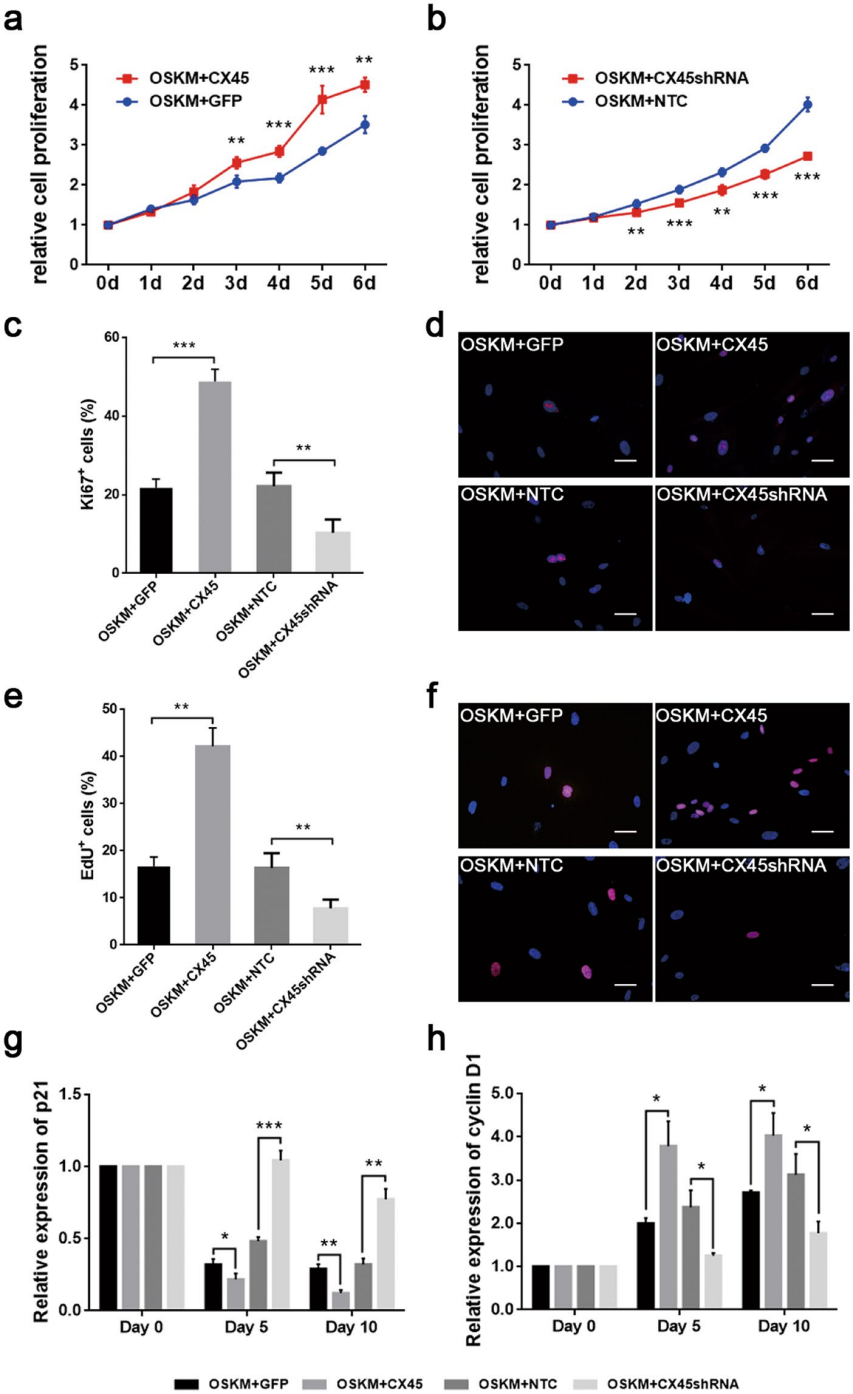
CX45 overexpression or knockdown affected the cell proliferation. (**a**,**b**) Cell proliferation on the indicated days was tested by CCK8 kit. Absorbance values of wells in day 0 were arbitrarily assigned a value of 1.0. (**c**) Percentage of proliferating cells (Ki67^+^ cells) out of total population. (**d**) Fluorescence micrographs show Ki67^+^ cells (red) in each group; the nuclei were visualised with DAPI staining (blue). (**e**) Percentage of proliferating cells (EdU^+^ cells) out of total population. (**f**) Fluorescence micrographs show EdU^+^ cells (red) in each group; the nuclei were visualised with DAPI staining (blue). (**g** and **h**) Real-time PCR analysis to detect the expression levels of p21 and cyclin D1 genes. Expression was normalized to GAPDH and gene expression on day 0 was arbitrarily assigned a value of 1.0. The data are presented as the mean ± S.E.M. (n = 3) and are representative of three independent experiments. *P < 0.05, **P < 0.01, ***P < 0.001 versus OSKM+GFP or OSKM+NTC group. OSKM+GFP, eGFP expression vector plus OSKM; OSKM+CX45, CX45 expression vector plus OSKM; OSKM+NTC, containing nonspecific shRNA plus OSKM; OSKM+CX45shRNA, containing CX45 shRNA target sequences plus OSKM.

During the reprogramming process, we found that cells ectopically expressing CX45 seem to proliferate more quickly than the control (Supplementary Fig. S4a). Cell proliferation was detected by CCK8 assay and the cell proliferation rate was significantly increased in OSKM+CX45 group and decreased in OSKM+CX45shRNA group. There was a significant difference in growth rate of cells between the OSKM+CX45 group and OSKM+GFP group at 3 days post reprogramming (Fig. 4a). The cell proliferation was inhibited when treated with CX45 shRNA lentivirus plus OSKM. Statistical results showed that there was prominent difference between OSKM+CX45shRNA group and OSKM+NTC group control at 2 days post reprogramming (Fig. 4b).

To confirm that CX45 overexpression increases cell proliferation during the reprogramming process, we then tested Ki67 expression. The results showed that 21.73 ± 2.25% of cells in control were Ki67-positive compared with that 48.80 ± 3.13% of cells when CX45 was overexpressed during reprogramming (mean ± S.E.M., Fig. 4c and d). When CX45 was repressed, the percentage of Ki67-positive cells was significantly decreased (10.30 ± 1.68%) compared with control group (22.20 ± 1.70%) (mean ± S.E.M., Fig. 4c and d). EdU assay allows the percentage of cells at S-phase to be determined. Subsequently, immunodetection of EdU was preformed, and the results showed that overexpression of CX45 significantly increased the percentage of EdU positive cells (42.13 ± 3.91%) and there was only 16.68 ± 1.98% in OSKM + GFP group (mean ± S.E.M., Fig. 4e and f). In contrast, knockdown of CX45 dramatically decreased the S-phase fraction of EdU incorporated cells from 16.35 ± 1.55% (OSKM + NTC group) to 7.75 ± 0.94% (OSKM + CX45shRNA group) (mean ± S.E.M., Fig. 4e and f). To further explore the underlying mechanisms, we tested the expression levels of several cell cycle-regulated genes. The expression level of p21 protein was significantly decreased while the expression level of cyclin D1 protein was enhanced in OSKM + CX45 group compared with OSKM + GFP group (Fig. 4g and h). When knockdown of CX45, the level of p21 protein was increased and cyclin D1 protein was decreased significantly by compared with OSKM + NTC group. Neither ectopic expression nor knockdown of CX45 significantly affected the expression levels of p53 and p27 protein (Supplementary Fig. S4b and c). Our data suggest that CX45 may increase the cell division rate, resulting in accelerated kinetics of iPSC production.

## Discussion

Here, we showed that CX45, a member of the CX family, played an important role in cellular reprogramming to a pluripotent stem cell. First, CX45 expression was enriched in hESCs and hiPSCs, but absent in hDFs. Second, CX45 protein level was dramatically upregulated during the reprogramming process. Thirdly, overexpression of CX45 enhanced the reprogramming of somatic cells to pluripotent state, while knockdown of CX45 repressed the reprogramming efficiency.

Many studies had demonstrated that iPSCs were highly similar but not completely identical to ESCs based on the analysis of gene expression profiles^26^. Assou et al. summarized microarray studies in hESCs of 38 previous reports and regarded CX43 and CX45 as the undifferentiated hESCs markers^27^. In our study, we found that the expression level of CX45 in hiPSCs and hESCs were not significantly different, however, CX45 was not detected in hDFs. In addition, the RNA sequencing data also revealed that the CX45 expression of hiPSCs was the same as of hESCs (Supplementary Table 1; GSE79928). We also observed CX45 involved functional GJIC formation in human iPS colonies through scrape loading/dye transfer assays and saw remarkable reduced dye transfer by the pharmacological/peptide gap junction inhibitors.

CX45 protein was dramatically upregulated during the reprogramming process. Previously reports had found that CX45, like CX43, was more enriched in undifferentiated ESCs than a range of somatic tissues or spontaneously differentiated hESCs^20, 21^. What is interesting was that we show here for the first time that the CX45 overexpression can significantly enhance the efficiency of iPSC generation, while the knockdown of endogenous CX45 expression remarkably reduced the reprogramming efficiency, suggesting an important role of CX45 in reprogramming. There was an increase in the number of TRA-1-60^+^ staining clones when CX45 was included in the reprogramming cocktail, which is a reliable marker for fully pluripotent iPSCs^28, 29^. Moreover, both the *in vivo* and *in vitro* analyses revealed that these CX45-hiPSCs exhibited the essential hESC-like characteristics of self-renewal and differentiation, and expressed the pluripotency markers and differentiated to tissues from all three germ layers, indicating that the addition of CX45 did not interfere with the pluripotent/differentiation potential of the iPSCs.

We had previously reported that ectopic expression of CX43 could increase the efficiency of iPSCs generation^30^. Sometimes, the expression of CX may be affected by the presence of other CXs through compensatory changes^31^, so we performed a western blotting analysis and found that CX45 did not affect the expression of CX43 (Supplementary Fig. S5). We inferred that CX45 may play a unique role in reprogramming that cannot be compensated by the presence of CX43. Our further investigation found that CX45 seem to promote the proliferation of cells. The ability of increased cell division rate to accelerate and drive the kinetics of the reprogramming process has been reported previously^32, 33^. Cell division was thought to amplify the number of daughter cells from partially reprogrammed cells where each resulting individual cell has an independent probability of progressing towards becoming an iPSCs. For example, LIN28 overexpression or vitamin C was thought to promote iPSCs formations in this way by inhibiting p53 and p21 expression, thus enhanced pre-iPSCs to iPSCs transition and increased reprogramming efficiency^34, 35^. Further, we found ectopic expression of CX45 could bring down the expression of p21 and upregulate the expression of cyclin D1, although it did not affect p53 and p27 expression well. Several studies have reported that iPSC generation was markedly enhanced by p53 suppression^36, 37^. In our study, experiments with the OSKM plus p53 shRNA with and without CX45 were performed respectively, and we found that p53 suppression plus CX45 could generate significantly higher number of iPSC colonies, which indicates that the p53 suppression and CX45 may have distinct roles during reprogramming (Supplementary Fig. S6).

Although p21 was first found to be directly induced by p53 and controlled cell proliferation in a p53-dependent manner^38^, numerous studies have reported that ERK1/2 is another essential factor in the regulation of p21. Chen et al. reported that HMGB1 promotes hepatocellular carcinoma progression partly by downregulating p21 via enhancing the ERK1/2^39^. Cao et al. also reported that ablation of TEM8 led to increasing of p21 and suppression of cyclin D1 by inhibited the activation of ERK1/2 in osteosarcoma cells^40^. On the other hand, Khodosevich et al. reported that overexpression of CX45 by retroviral injections increased the proliferation of Mash-1-positive transit-amplifying precursor cells in the SVZ and CX45 positively influenced cell cycle via activating ERK1/2 pathway by evoke Ca^2+^ release from intracellular stores and extracellular Ca^2+^ influx^41^. Overall, CX45 increases the efficiency of reprogramming, in part, by accelerating individual cell division rate.

There are some limitations of this study. First, non-integrative method is thought to be the best way for reprogramming human cells due to its high safety and feasibility^34, 37^. Okita et al. reported a simple non-integrative method using p53 suppression and non-transforming L-MYC with episomal plasmid vectors for reprogramming human cells^37^. Using a non-integrative system to ectopic expression CX45 for cell reprogramming needs further investigation. Second, ectopic overexpression of a new gene during reprogramming may increase tumorigenicity of iPSCs. Reikvam et al. reported that CX45 expression show a wide variation between human myeloid leukemia cells at the mRNA level and a high CX45 expression was associated with the altered regulation of the mitogen-activated protein kinase (MAPK) pathway, whereas a low CX45 expression was associated with the altered regulation of protein functions^42^. While in another study, it is reported that CX45 expression was reduced in colorectal carcinomas compared to normal tissue samples^43^. Although CXs are mostly suggested to act as tumor suppressor genes in various tissues, the safety of CX45 overexpression during reprogramming still needs further study.

In conclusion, we first demonstrate that CX45 is highly enriched in hDFs-derived undifferentiated hiPSCs during and after the reprogramming process but absent in hDFs. In addition, CX45 overexpression enhances the reprogramming and the cell proliferation, while knockdown of CX45 turns out contrary. Those may function by inhibiting the p21 and cyclin D1 expression. Together, our findings contribute to the understanding the underlying mechanism of somatic reprogramming and assist in the exploitation of iPSC technology.

## Materials and Methods

### Cell culture

HES cell line (H1) is used as control pluripotent cell lines. H1 and the hiPSCs were maintained on either MEF feeders or in feeder-free culture conditions using standard methods^44, 45^. The ES medium consisted of knockout DMEM (Invitrogen) supplemented with 15% (v/v) knockout serum replacement (Invitrogen), 5% (v/v) fetal bovine serum (FBS), 0.1 mM beta-mercaptoethanol (Sigma), 1% (w/v) non-essential amino acid (Hyclone), 1% (w/v) penicillin/streptomycin, 2 mM L-glutamine and 10 ng/ml human basic fibroblast growth factor (Chemicon). To allow feeder-free growth of H1 and hiPSCs, plates were coated with 0.3 mg/ml Matrigel (BD Biosciences) and cultured in mTeSR1 medium (Stem Cell). The colonies were harvested by treatment with dispase (Invitrogen). 293FT cells and hDFs were cultured in DMEM containing 10% (v/v) FBS, 2 mM L-glutamine (Invitrogen), 1% (w/v) non-essential amino acids (Hyclone) and 1 mM sodium pyruvate (Sigma). HDFs were isolated from a foreskin biopsy of a healthy volunteer as previously described^45^ with informed consent.

### Ethics Statement

All research protocols for this study were approved by the Ethics Committee of Sun Yat-sen University (Guangzhou, China). Informed consent was obtained from healthy volunteer prior to obtaining specimens. All samples were treated as stated in the approved ethical application and the research methods were carried out adhering to the relevant guidelines. All of the animal experimental procedures were approved by the Animal Ethics Committee of Sun Yat-sen University and the detail methods were given in the supplementary material.

### Western blotting

Cell lysates were separated via SDS–PAGE using 8% (w/v) Tris-glycine mini-gels and transferred to polyvinylidene difluoride membranes as previously described^46^. The primary antibodies used were as follows: rabbit anti-CX45 (1:1 000; MAB3100; Millipore), rabbit anti-NANOG (1:2 000; D73G4; Cell Signaling), mouse anti-CX43 (1:2000; C8093; Sigma), and mouse anti-beta-TUBULIN (1:10 000; T4026; Sigma). After incubation with enhanced chemiluminescence (Amersham), immunopositive bands were visualized and scanned with a GS-800 Calibrated Imaging Densitometer (Bio-Rad). All of the western blotting exposures were within the linear detection range. The intensity (area × density) of the individual bands on western blotting was measured by a Quantity-One software (Bio-Rad) and the amount of target protein was normalized to β-TUBULIN.

### Real-time PCR

Total RNA was purified with the RNeasy Plus Mini kit (Qiagen) according to the manufacturer’s instructions. The concentration and quality of total RNA samples were checked by a NanoDrop ND-1000 (NanoDrop) and one microgram total RNA was reverse-transcribed using an AMV First-Strand cDNA synthesis kit (Invitrogen). A real-time quantitative PCR was performed using the SYBR qPCR mix (Toyobo) and a Roche Light Cycler 480 PCR instrument. A real-time quantitative PCR was performed under the following conditions: 5 min at 94 °C, 40 cycles of 15 s at 94 °C, 15 s at 58–61 °C, and extension at 72 °C for 40 s. GAPDH was used as an internal control to normalize the other genes. All of the PCR primers were tested and only the samples with single and matching end-point melting curve peaks were used for subsequent analysis and some of them were designed according to a previous report^47^. The primer sequences used in this paper were show in Supplementary Table 2.

### Scrape Loading/Dye Transfer Assay

Scrape loading/dye transfer assay was performed to assess GJIC activity as previously described^30, 48, 49^. Cells were rinsed three times with prewarmed phosphate-buffered saline (PBS) and then treated with CBX (carbenoxolone, 100 μM; 4 h; Sigma), 18-α-GA (18-α-glycyrrhetinic acid, 25 μM; 4 h; Sigma), or the synthetic peptide (amino acid sequence QVHPFYVCSRLPCPHK, 300 μM; 4 h; CPG Biotech) homologous to CX45 at 37 °C for 3 h. A mismatched peptide (amino acid sequence EIKKFKYGC, 300 μM; 4 h; CPG Biotech) served as a negative control. Then the cell colonies were scraped and incubated for 5 min with Lucifer yellow (2 mg/ml, Sigma) and rhodamine-dextran (2 mg/ml, Invitrogen) diluted in PBS. Dye transfer can be observed by fluorescence microscopy, and the distance between the dye transfer front and the scrape line was analysed.

### Immunofluorescence staining

To measure immunofluorescence, all cells were fixed with 4% (v/v) paraformaldehyde in 0.1 M PBS (pH 7.4). Before staining, the cells were treated with 0.1% (v/v) Triton X-100 and blocked using 2% (w/v) bovine serum albumin. And then, the cells were incubated at 4 °C overnight with the primary antibody: anti-NANOG (1:300; Cell Signaling), anti-OCT4 (1:100; Santa Cruz), anti-SSEA-4 (1:100; DSHB), anti-TRA-1-60 (1:100; Millipore), anti-ki67 (1:50; eBioscience) and anti-Cx45 (1:300; Millipore). Appropriate Alexa 488 and Alexa 555 labeled secondary antibodies (Invitrogen) were used for visualization. The nuclei were counterstained with DAPI (4,6-diamidino-2-phenylindole; Sigma-Aldrich).

### Expression lentiviral vector construction

As previously reported, the entry clone containing the 1191 bp human CX45 (Fulengen) ORF (pDown-CX45) were cloned into pDest-puromycin by the Gateway LR reaction with pUp-EF1a and pTail-IRES-eGFP to generate the expression lentiviral vector, which is designated as pFinal/PGK-puro-EF1a-CX45-IRES-eGFP (abbreviated as CX45, Supplementary Fig. S1a). The pFinal/PGK-puro-EF1a-eGFP lentiviral vector (abbreviated as GFP) acted as a control.

To knock down CX45 and p53 synthesis, four short-hairpin RNAs (shRNA) designed were annealed and cloned into the pLL3.7 lentiviral vector (a kind gift from Dr David L. Garbers, University of Texas Southwestern Medical Center, Dallas, TX, USA). They were named CX45shRNA1, 2, 3 and p53shRNA. All the target sequences were showed in Supplementary Table 3.

### Reprogramming and viral infection

The above expression lentiviral vectors and retroviral vectors (OCT4, SOX2, KLF4 and c-MYC) were transfected into 293FT cells in the presence of packaging plasmids using X-tremeGENE HP DNA Transfection Reagent (Roche), respectively. After 48–72 h, the supernatants were harvested and concentrated by ultracentrifugation (50 000 g; 120 min at 4 °C). HDFs were transduced with each lentivirus plus OSKM with 8 μg/ml polybrene (Sigma). Each culture medium was replaced 12 h after infection. After a further 12 h, the hDFs were re-infected, and the culture medium replaced again; “day 0” commenced at this time. The enhancement or knockdown efficacy of each lentivirus was assessed by western blotting. Around day 5, samples of cells were transferred in ES medium to 6-well plates bearing irradiated feeder layers at a density in the range of 1 × 10^4^ to 5 × 10^4^ cells per well. Colonies were calculated for efficiency evaluation or selected for establishment of cell lines. TRA-1-60-positive colonies were counted by three independent experiments for each group. TRA-1-60 expression (1:100; Millipore) on colonies was analysed with immunocytochemistry using the Vectastain ABC Kit (lgM) (Vector lab) to identify fully reprogrammed iPSC colonies.

### Cell Proliferation Assay and EdU Detection

The cell proliferation was measured by Cell Counting Kit-8 detection kit (CCK-8, Dojindo). After infected, HDFs were seeded at a concentration of 5 × 10^3^ cells per well in 96-well plates. CCK-8 solution was applied at 10 μl per well and followed by 3 h incubation at 37 °C every day. Absorbance values of all wells were then determined at 450 nm in Microplate Reader (Bio-Rad) represented relative to the control values. All experiments were conducted in triplicate.

To label proliferating cells in the reprogramming process, hDFs were treated with EdU overnight at a concentration of 10 μM at day 5. And then cells were fixed and permeabilized followed by EdU detection according to the manufacturer’s instructions. The number of proliferating cells in the reprogramming process that reenter cell cycle was determined as the percentage of EdU-positive cells in the DAPI-positive cell population.

### Statistical analysis

All of the data were statistically examined using the unpaired Student’s *t*-test; Significance was assumed at *P* < 0.05, and all P-values are presented as two-tailed. The data are presented as the means ± S.E.M. calculated using the SPSS software (version 17.0).

## Electronic supplementary material

Cx45 supplementary figure legends and tables
