# Supplementary material for: Enhanced generation of human induced pluripotent stem cells by ectopic expression of Connexin 45
Source: Sci Rep. 2017 Mar 28;7:458. doi: 10.1038/s41598-017-00523-y (PMC5428559; doi:10.1038/s41598-017-00523-y)
Supplement: Supplementary file 1 — Cx45 supplementary figure legends and tables [file 41598_2017_523_MOESM1_ESM.pdf]

Title: Enhanced generation of human induced pluripotent stem cells by ectopic expression of Connexin 45

Author： Qiong Ke, Li Li, Xin Yao, Xingqiang Lai, Bing Cai, Hong Chen, Rui Chen, Zhichen Zhai, Lihua Huang, Kai Li, Anbin Hu, Frank Fuxiang Mao, Andy Peng Xiang, Liang Tao, Weiqiang Li

Corresponding author：

WQ Li: Sun Yat-Sen University, Guangzhou, China.

E-mail: liweiq6@mail.sysu.edu.cn. Tel: +8620-87335982.

L Tao: Sun Yat-Sen University, Guangzhou, China.

E-mail: taol@mail.sysu.edu.cn. Tel: +8620-87332318.

Supplementary Figure S1 Vector construction and transduction efficiency of lentiviruses

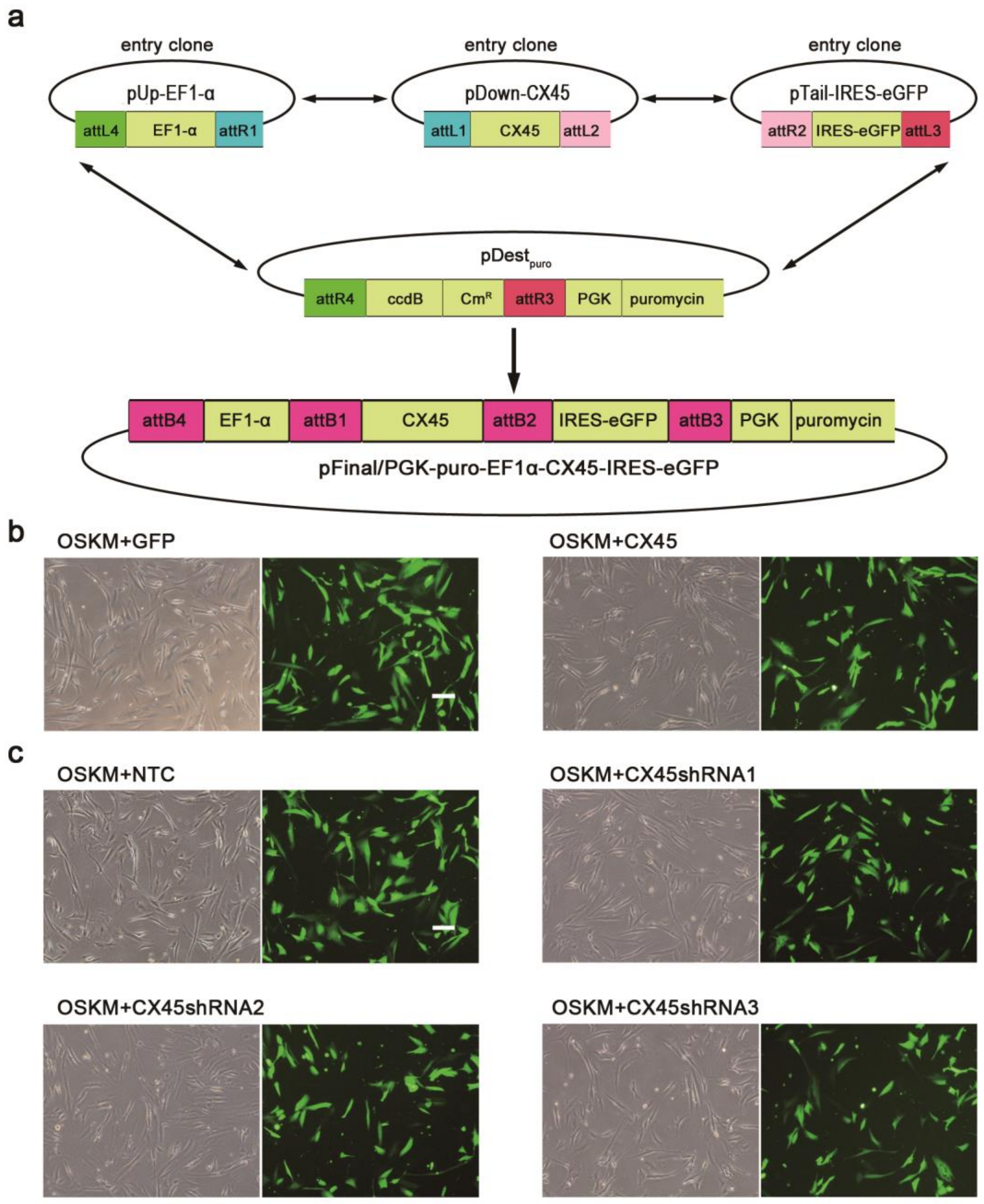

(a) A schematic diagram of the construction of the pFinal/PGK-puro-EF1α-CX45-IRES-eGFP vector (abbreviated as CX45). (b-c) The phase-contrast and fluorescence micrographs show the GFP protein expressed in the transduced hDFs 48 h after transduction. Scale bar = 200 μm. OSKM+GFP, eGFP expression vector plus OSKM; OSKM+CX45, CX45 expression vector plus OSKM; OSKM+NTC, containing nonspecific shRNA plus OSKM; OSKM+CX45shRNA1, 2 and 3, containing CX45 shRNA target sequences plus OSKM.

Supplementary Figure S2 Ectopic expression CX45 with different combinations of reprogramming factors different transcription factors promotes the reprogramming efficiency

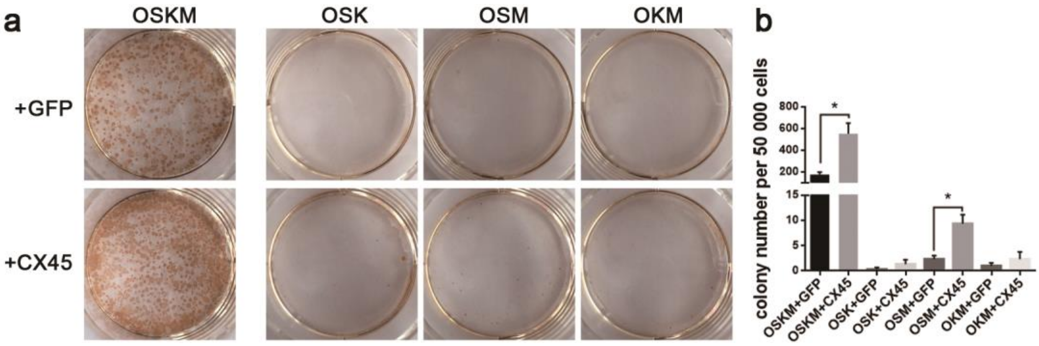

(a) Representative images of TRA-1-60-positive (TRA-1-60$^+$) colonies obtained with different combinations of reprogramming factors. (b) Quantification of TRA-1-60$^+$ colonies per 50 000 cells as indicated. The data are presented as the mean ± S.E.M. (n=3). *P<0.05, **P<0.01. GFP, eGFP expression vector; CX45, CX45 expression vector.

Supplementary Figure S3 Characterization of hiPSCs derived from hDFs by OSKM plus CX45 (CX45-hiPSCs)

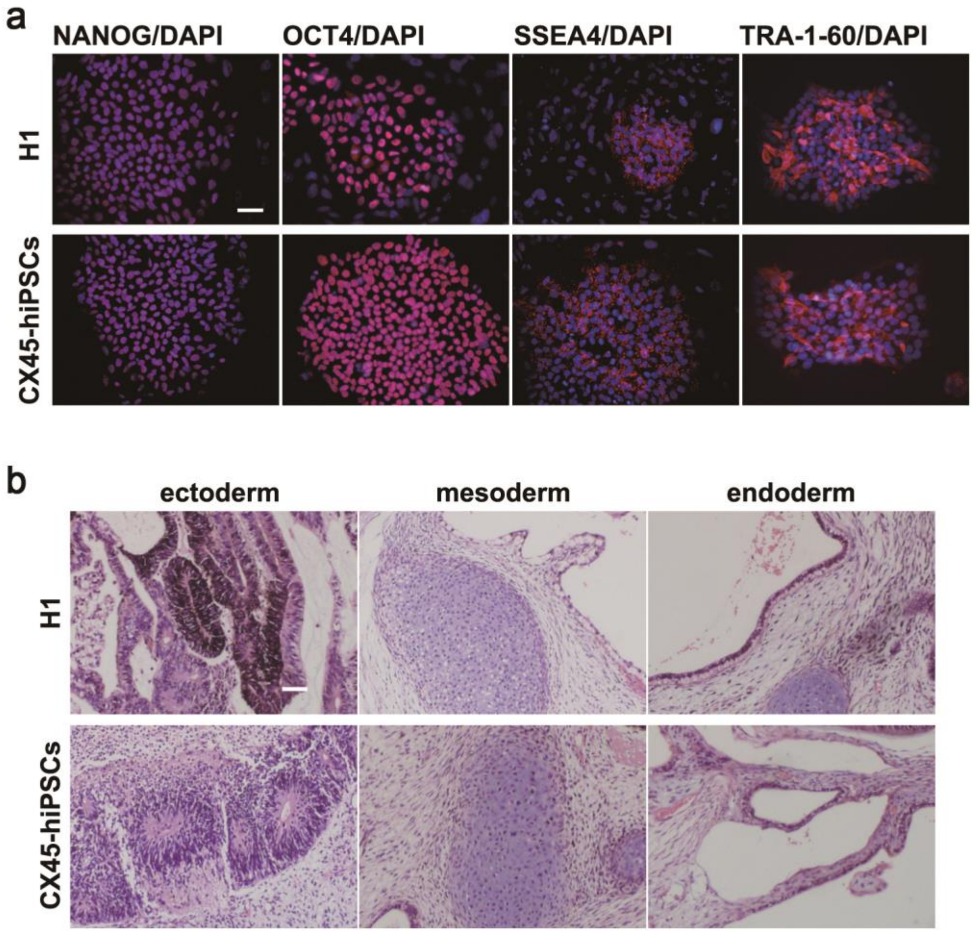

(a) Immunofluorescence analysis of the indicated pluripotent markers in CX45-hiPSCs. (b) The CX45-hiPSCs formed teratomas in vivo, and H&E staining of teratoma sections showed all three germ layers (endoderm-derived glandular epithelium, mesoderm-derived cartilage and ectoderm-derived neural tubes). Scale bar = 50 μm.

For teratoma production, approximately 1×10^{7} human iPS cells were suspended in DMEM and injected subcutaneously into nude mice. Teratomas were dissected after 2 months and fixed overnight in 4% (v/v) paraformaldehyde. Paraffin-embedded tissues were sliced and subjected to H&E staining to detect in vivo differentiation. All of the animal experimental procedures were approved by the Animal Ethics Committee of Sun Yat-sen University.

Supplementary Figure S4 CX45 overexpression or knockdown affected the cell proliferation

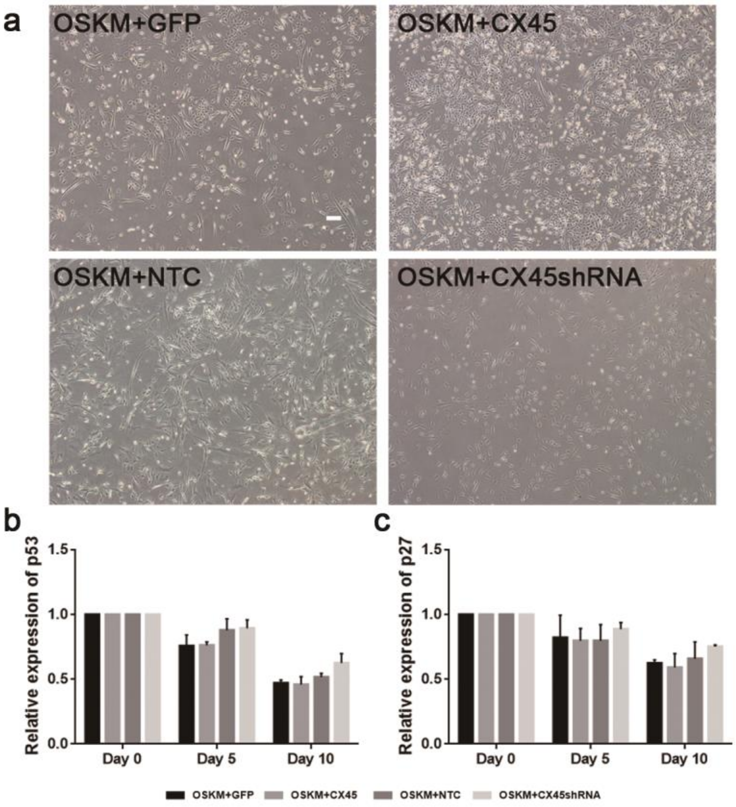

(a) The phase-contrast showed the proliferation of hDFs on day 6 after transduction. Scale bar = 200 μm. (b) and (c) Real-time PCR analysis to detect the expression levels of p53 and p27 genes. The transcript levels were normalised to the levels of cells on day 0. The data are presented as the mean ± S.E.M. (n=3) and are representative of three independent experiments. OSKM+GFP, eGFP expression vector plus OSKM; OSKM+CX45, CX45 expression vector plus OSKM; OSKM+NTC, containing nonspecific shRNA plus OSKM; OSKM+CX45shRNA, containing CX45 shRNA target sequences plus OSKM.

Supplementary Figure S5 Ectopic expression of CX45 does not affect CX43

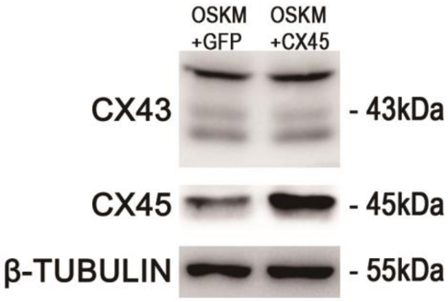

Ectopic expression of CX45 does not affect CX43 expression during the reprogramming process on day 5. OSKM+GFP, eGFP expression vector plus OSKM; OSKM+CX45, CX45 expression vector plus OSKM.

Supplementary Figure S6 Ectopic expression CX45 combined with p53 suppression promotes the reprogramming efficiency

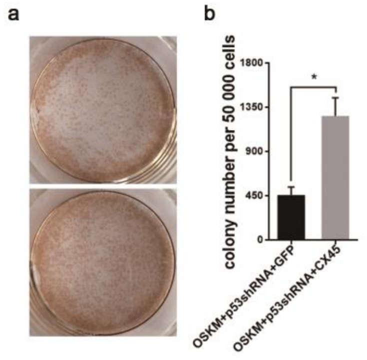

(a) Representative images of TRA-1-60$^+$ colonies obtained with different combinations of reprogramming factors. (b) Quantification of TRA-1-60$^+$ colonies per 50 000 cells as indicated. The data are presented as the mean ± S.E.M. (n=3). *P<0.05. p53shRNA, p53 shRNA target sequences; GFP, eGFP expression vector; CX45, CX45 expression vector.

Supplementary Table 1. RNA sequencing data of Connexins.

GeneName	H1	hiPSCs
GJA1 (CX43)	302.5828	326.8701
GJA10 (CX62)	0.0417	0
GJA3 (CX46)	0.3922	0.962
GJA4 (CX37)	0.5454	0.4229
GJA5 (CX40)	0.9703	0.5643
GJA8 (CX50)	0	0
GJA9 (CX59)	0	0
GJB1 (CX32)	0	0
GJB2 (CX26)	0.1459	0.0588
GJB3 (CX31)	0.1545	0.4565
GJB4 (CX30.3)	0.1438	0.0725
GJB5 (CX31.1)	1.0625	0.4284
GJB6 (CX30)	0.0714	0
GJB7 (CX25)	0.6865	1.1733
GJC1 (CX45)	16.7525	19.7157
GJC2 (CX47)	0.3034	0.6728
GJC3 (CX30.2)	1.0976	0.8605
GJD2 (CX36)	0.2818	0
GJD3 (CX31.9)	1.5493	1.324
GJD4 (CX40.1)	0	0

Supplementary Table 2. The primers used in this work.

Cx45-F	5’-GAGCTTCCTGACTCGCCTGCT-3’
Cx45-R	5’-CCCGGCTGTTCTGTGTTGCAC-3’
P21-F	5’-GGAAGACCATGTGGACCTGT-3’
P21-R	5’-GGCGTTTGGAGTGGTAGAAA-3’
P27-F	5’-TGCAACCGACGATTCTTCTACTCAA-3’
P27-R	5’-CAAGCAGTGATGTATCTGATAAACAAGGA-3’
Cyclin D1-F	5’-AACTACCTGGACCGCTTCCT-3’
Cyclin D1 -R	5’-CCACTTGAGCTTGTTCACCA-3’
P53-F	GCTCGACGCTAGGATCTGAC-3’
P53-R	5’-AGCTTCTGAATGCTTTTTGGG-3’
GAPDH-F	5’-AATCCCATCACCATCTTCCAGGAG-3’
GAPDH-R	5’-CACCCTGTTGCTGTAGCCAAATTC-3’

Supplementary Table 3. The shRNA target sequence used in this work.

CX45shRNA1	5’ GGAGGAGAAUCCAUCUAUU dTdT 3’
CX45shRNA2	5’ GGACCAUUCGAGACUCACU dTdT 3’
CX45shRNA3	5’ CCGAACUGUCCAAUGCUAA dTdT 3’
p53shRNA[50]	5’ TTTCATCAGCAAGTTTGGA dTdT 3’

Reference

50. Masutomi, K. et al. Telomerase maintains telomere structure in normal human cells. *Cell* **114**, 241-53 (2003).
